# Layperson-Oriented versus Clinical-Based Models for Assessing 10-Year Incidence of Coronary Heart Disease: National FINRISK Study

**DOI:** 10.1155/2011/823782

**Published:** 2011-10-19

**Authors:** Qing Qiao, Tiina Laatikainen, Weiguo Gao, Janne Pitkäniemi, Erkki Vartiainen

**Affiliations:** ^1^Department of Public Health, University of Helsinki, PL41 Mannerheimintie 172, 00014 Helsinki, Finland; ^2^Department of Chronic Disease Prevention, National Institute for Health and Welfare, P.O. Box 30, 00271 Helsinki, Finland

## Abstract

One laboratory-based and two non-laboratory-based models with and without blood pressure measures are developed based on data of 14815 men and 16617 women aged 25–64 years. During the followup 1134 men and 566 women developed coronary heart disease (CHD). The area under the receiver operating characteristic curve (95% CI) for prediction of CHD incidence was 0.823 (0.807–0.839) for the laboratory-based model, 0.808 (0.791–0.824) and 0.803 (0.787–0.820) for the non-laboratory-based models with and without systolic blood pressure in men (*P* < 0.01 for overall comparison), and 0.878 (0.856–0.901), 0.871 (0.848–0.894), and 0.864 (0.840–0.887), respectively, in women (*P* < 0.01). The predicted rates matched well with the observed ones (*P* > 0.10). Compared with the model without blood pressure, the non-laboratory-based model with blood pressure tended to reclassify individuals into the higher risk categories for both event and nonevent groups in both genders. The study concludes the predictive property of the non-laboratory-based models are good.

## 1. Introduction

Beginning 50 years ago, the Framingham Heart Study forever changed our approach to predict and prevent coronary heart diseases by identifying major risk factors for myocardial infarction (MI) [1–4] and developing risk assessment function based on the major risk factors [5, 6]. Since then many other cardiovascular diseases (CVDs) risk prediction functions or scores have been developed [7–12]. All these previous prediction models require laboratory and clinical examinations and target at high-risk individuals who visit a clinic. A simplified risk assessment tool is, however, required for CVD prevention through population approach that aims to reduce the risk factors at population levels through lifestyle and environment changes. Such a simplified tool is also useful in less developed countries where the health care resources are limited but the largest increase in CVD will occur [13]. Recently, an American study showed that a model that uses non-laboratory-based risk factors predicted CVD events as accurately as one that relied on laboratory-based values [14]. This has, however, not been confirmed yet by other studies. Based on data of the National FINRISK Study, we developed a model which does not include laboratory variables and blood pressure measurement and compared it to the models which includes these measurements with regard to their predictive ability of first events of coronary heart disease (CHD).

## 2. Methods

The National FINRISK Study has been carried out every 5th year since 1972 to monitor CVD risk factor levels and evaluate health strategies to lower CVD events in Finland [15] based on random population representative samples. Cohorts first examined in 1982, 1987, 1992, 1997, and 2002, including about 14815 Finnish men and 16617 Finnish women aged 24–64 years, were followed up using national registers until 31 December of 2007. The maximum follow-up length was 5.9 years for the 2002 cohort and 25.9 years for the 1982 cohort. The survey methods followed the standard protocol (http://www.ktl.fi/ehrm), which have been described in details elsewhere [15].

### 2.1. Ascertainment of the CHD Events

First ever CHD events were ascertained through computerized record linkage of the unique national ID numbers of the survey participants to the national Causes of Death Registry and the national Hospital Discharge Registry. International Classification of Disease (ICD), 9th revision (10th revision), was used for the classification of either the fatal or the nonfatal events. ICD codes of 410–414 (I20–I25) for fatal CHD and 410-411 (I21-I22, I24) for nonfatal acute MI were applied. The first nonfatal MI and the first fatal CHD events constitute the incident CHD events. Subjects with a self-reported history of ischemic heart diseases or ascertained from the registers as having ischemic heart diseases before the baseline survey of each study cohort were excluded from the data analysis.

### 2.2. Statistical Analysis

Cox proportional hazard model analyses were performed to relate factors to the first ever CHD events using STATA 11.0. The assumption of the proportionality was tested based on Schoenfeld residuals (phtest) method for each explanatory variable for each cohort and for men and for women. The results showed the assumption of the proportionality of hazards holds; *P* values of the global tests varied from 0.20 to 0.93. 

#### 2.2.1. Selection of Risk Predictors

50 candidate variables, including age, marital status, spouse and children, self-evaluation of one' personal or family life, education and occupation, stress from work and family, family income, dietary components, physical activities at work and leisure time, tobacco and alcohol consumption, self-consideration of one's health status and functional capacity, history of diabetes or hypertension or elevated total cholesterol, parents' history of cardiovascular diseases, body mass index (BMI), and laboratory measurements of total cholesterol and HDL-cholesterol, ratio of total cholesterol to HDL cholesterol (cho/HDL), were examined in either univariable or stepwise multivariable models. A variable list with detailed variable description is presented in the supplemental digital Table  1 in supplementary material available online at doi: 11.55/2011/823782. Age, residential areas, survey years, and years of education were fitted in all models. Cohort specific baseline survival function (or rate) at the mean values of risk factors of the whole study population was estimated. All the continuous variables were logarithmically transferred to improve discrimination and calibration of the models and to minimize the influence of extreme observations.

#### 2.2.2. Three Prediction Models and CHD Risk Assessment Function

Three different Cox models were finally constructed: one laboratory based and two non laboratory based. Both the chol/HDL and the systolic blood pressure entered into the laboratory based model. BMI did not enter into the laboratory based model, but into the non-laboratory-based models at the absence of the chol/HDL. Chol/HDL was removed from the laboratory-based model to form the non-laboratory-based models, and systolic blood pressure was further taken out from the non-laboratory-based model to form the layperson-oriented models.

The regression coefficient of each factor was estimated based on pooled data of all five cohorts, but the cohort-specific baseline survival rates at 10 years derived from the Cox models were used to calculate the 10-year absolute risk for each cohort using the formula described in the Framingham Heart Study [16].

#### 2.2.3. Assessment of the Model Performance and Comparison between Models

**Discrimination**

Receiver operating characteristic (ROC) curves of the 10-years absolute CHD risk calculated based on the estimates of the three different Cox models were plotted. Areas under the curves (AUCs) were calculated to evaluate discrimination of the models, and the differences in AUCs were tested based on C statistic.

**Calibration**

Calibration, a measure of agreement between observed and predicted CHD events within 10 years, was made for the layperson-oriented model. The 10-year absolute CHD risk was used to divide individuals into deciles of the predicted risk. The mean of the predicted probability of having the CHD in each decile and the observed probability obtained using Kaplan-Meier method was plotted. The difference between observed and predicted rate was tested using *χ* ^2^-test with 9*df*.

**The Net Reclassification Improvement (NRI)**

NRI was calculated according to Pencina et al. [17] to quantify the improvement in the right reclassification of the layperson-oriented model against the other two models in either event or non-event groups. According to the guidelines for CVD prevention from different major organizations or study groups based on 10-year absolute risk assessment [18–20], 10-year CHD risk categories of <5%, 5%–9%, 10%–19%, and ≥20% were made. If a model moves a CHD event from a low risk category to a high one, the reclassification was considered right and the mode prediction was improved compared with the reference model. A downgrade for the non-events was considered as right reclassification.

## 3. Results

Characteristics of the cohorts are shown in Table 1. BMI and the prevalence of diabetes have increased while blood pressure and unfavourable lipid profiles decreased during the last 35 years in Finland as reported [15]. 

Altogether 1131 (7.6%) first ever CHD events in men and 561 (3.4%) in women were observed during the followup. Individuals who developed CHD events were older and had higher BMI, blood pressure and worse lipid profiles than those who were free of CHD at the end of the followup (Table 2). The proportion of people with prior history of diabetes or hypertension, parent(s) suffering from MI, and self-perceived worse health conditions were also higher in the former than in the latter. Current smoking in men and lower education level in both genders were related to incidence of CHD. 

The regression coefficients of the predictors and the baseline survival function for each cohort are shown in Table 3. The estimated 10-year mean absolute CHD risk were 3.76%, 3.85%, and 3.76% in men for the laboratory based model, the non-laboratory based models with and without blood pressure; they was 1.25%, 1.37%, and 1.33%, respectively, in women. Given a positive test at the risk of ≥20%, the positive predictive values for 10-years CHD incidence were similar between the models (22.4% in men and 25.0% in women by the layperson-oriented model, 20.2% and 25.6% by the laboratory-based model and 21.2% and 21.8% by the non-laboratory-based model with systolic blood pressure). However, the number of individuals referred to further tests or intervention was largest for the laboratory-based model (*n* = 286) than for the non-laboratory based models with (*n* = 258) and without (*n* = 209) blood pressure measurement. 

**Figure 1:**
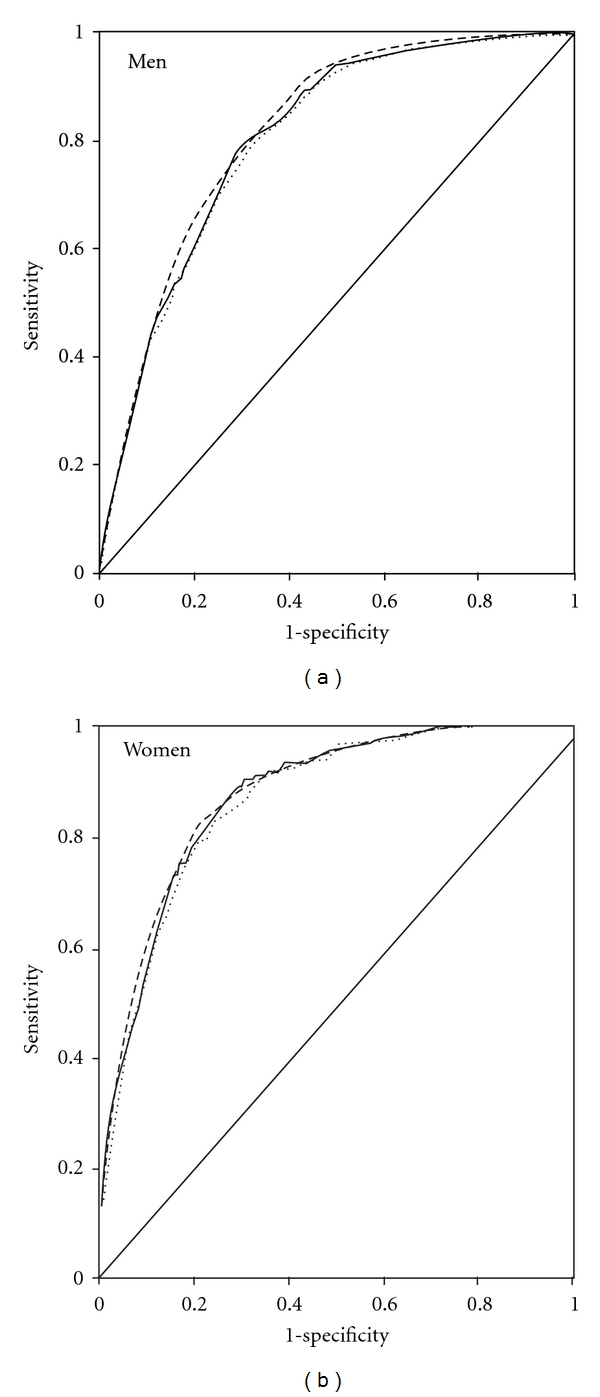
Receiver operating characteristic curves for 10-year incidence of coronary heart disease predicted by the laboratory-based model (dashed), non-laboratory-based model (solid) and the layperson-oriented model (round dot).

 All three predictive models adequately discriminated people with CHD events from those without as shown in Figure 1 (the ROC curves). The AUC (95% CI) was 0.823 (0.807–0.839) for the laboratory-based model, 0.808 (0.791–0.824) and 0.803 (0.787–0.820) for the non-laboratory-based models with and without systolic blood pressure in men, and 0.878 (0.856–0.901), 0.871 (0.848–0.894), and 0.864 (0.840–0.887), respectively, in women. The layperson-oriented model gave slightly smaller AUC than others (*P* < 0.001 for overall comparisons in both genders).

**Figure 2:**
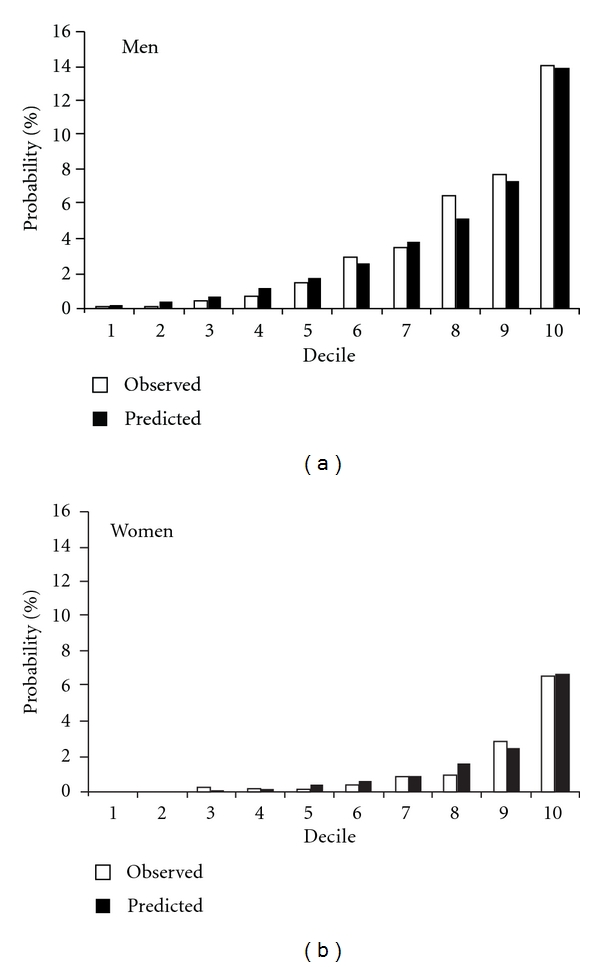
Observed (Kaplan-Meier, white) and predicted (black) probability of coronary heart disease in 10 years in deciles of risk estimated by the layperson-oriented model.

Calibration of the layperson-oriented model showed that the predicted 10-year event rate matched well with the observed event rate across deciles of the predicted risk in men (*χ* ^2^ = 14.15, 9*df*, *P* = 0.117) and in women (*χ* ^2^ = 12.36, 9*df*, *P* = 0.194) (Figure 2). Additional calibration was made for each of the five study cohorts at 5, 10, 15, 20, and 25 years, respectively, for cohorts 2002, 1997, 1992, 1987, and 1982. The predicted probability of having CHD events at 5, 10, 15, 20, and 25 years was close to those observed at each of the 6 risk categories as shown in supplemental digital Figure S1. 

Table 4 shows the net reclassifications of individuals with and without events across 10-year risk categories based on different models. The laboratory-based model correctly reclassified individuals in both event and non-event groups, but the overall NRI was significant only in men (8.1%, *P* = 0.001) not in women (2.9%, *P* = 0.432) as compared with the layperson-oriented model. Addition of the systolic blood pressure to the layperson-oriented model upgraded both events and non-events, with an overall NRI of 3.8% (*P* = 0.053) in men and 7.1% (*P* = 0.017) in women. 

The ROC curves and the NRI for two additional models that do not contain either the history of diabetes or hypertension are shown in supplemental digital Figure S2 and Table  2 in Supplementary Material available online at doi:11.55/2011/823782. The predictive ability of the models without either of the two was not impaired substantially in terms of discrimination and reclassification of individuals.

## 4. Discussion

The performance of the two non-laboratory-based CHD risk assessment models is good in terms of discrimination and calibration. Addition of the lipid measurements to the non-laboratory-based model slightly improved the reclassification of individuals in men but not in women, whereas addition of the systolic blood pressure into the layperson model improved the reclassification of individuals with the events but overestimated the risk in individuals without events. 

Laboratory tests and blood pressure measurement have been required in most previous prediction models [5–12, 16]. A recent study based on the National Health and Nutrition Examination Survey has, however, challenged the traditional way of building the models by showing that laboratory based model including total cholesterol performed no better than the non-laboratory based model requiring BMI rather than total cholesterol for predicting CVD outcomes [14]. Our current study lent further support to the approach to simplify the risk prediction model. Moreover, we show that a non-laboratory-based model not requiring measurement of the systolic blood pressure performed no worse than that requiring regarding to the prediction of the 10-year CHD incidence. The advantage of the simplified model over the traditional ones is that it does not require a clinic visit, and therefore, is both time and cost saving. This is important for CVD prevention using population approach and in the countries where the medical care resources are short and blood pressure measurement is not widely available. In spite of that the guidelines for CVD prevention have been produced by different organizations and study groups [18–20], most developing countries do not have a systematic screening program for CVD. A doctor has no obligation to estimate a patient's future CVD risk according to the patient's current health status. Thus there is no additional information rather than the complaint disorders will be discussed and handled during a hospital visiting. The layperson-oriented model as a supplemental tool may promote CVD risk screening and prevention by raising the public awareness of the disease through population approach, which is beyond the original intention to apply a CVD risk prediction score in a clinic setting targeting at high-risk individuals. To include BMI in the model is particularly important in public health considering the recent increase in obesity and diabetes worldwide, and can inform lifestyle changes. “Incapable to walk 500 meters without rest” appeared to be a strong early sign of the future CHD in spite of the causes of the inability. Thus, either a single component of the model or the integrated risk score estimated based on the model may serve as a trigger leading to early diagnosis and treatment of the CHD as well as other comorbid conditions such as hypertension, dyslipidemia and hyperglycemia. 

It needs, however, to be borne in mind that the layperson-oriented model derived from the Finnish population has not been externally validated and might need calibration before it is applied to other populations considering differences in CVD risk among countries. In spite of the differences in absolute risks, relative risks of certain risk factors such as smoking are relatively less variable across countries and age groups [21]. A risk score based on estimate of relative risk has, thus, been suggested but its predictive property needs to be further investigated against the one based on estimate of the absolute risk. 

Strengths of the current study include its large sample size, population-based study design, standard survey protocols and questionnaire, reasonable participation rates [15] and complete followup with first ever hard CHD events, including both fatal and nonfatal events. The Finnish Causes of Death register and Hospital Discharge register, the source of the data on CHD events in this study, have high coverage and diagnostic accuracy as reported previously [22]. The weakness of the FINRISK study is that the individual's risk factors were measured only at the baseline examinations and the changes in individual's risk factor levels during the followup are not known. Considering the decline in CHD risk and changes in risk factors in the population level in Finland during the last 35 years [15], the birth cohort-specific baseline survival function calculated at the mean values of covariates of the whole study population of all cohorts was used to calculate the absolute CHD risk of an individual person. The risk prediction function developed in this way has been shown to fit the data of individual cohort well even though of the cohorts were recruited over a 25-year period. 

In conclusion, the predictive ability of a layperson-oriented model is similar to the models including laboratory and systolic blood pressure measurements, but it is simple, noninvasive, time and cost saving, easy to use and has great potential to be applied in CVD prevention campaign through population approach.

##  Conflict of Interests

The authors have no conflict of interests.

## Supplementary Material

Figure S1. Observed (Kaplan-Meier, white) and predicted (black) probability of coronary heart disease at 5, 10,15, 20 and 25 years of follow-up in each of the six categories of the risk estimated by the layperson-oriented model.
Figure S2. Receiver operating characteristic curves for 10-years incidence of coronary heart disease predicted by the layperson-oriented model (blue, AUC=0.803 for men and 0.864 for women), layperson-oriented model without a history of diabetes (green, AUC=0.799 for men and 0.856 for women) or hypertension (red, AUC=0.801 for men and 0.856 for women). The AUC was slightly reduced when the history of diabetes was removed from the layperson model (*p*=0.04 for both men and women). AUC: Area under the curve.
Supplementary Table 1. Variables recorded at the baseline examinations and evaluated in the Cox regression analysis in the current study.
Supplementary Table 2. Reclassifications of individuals based on layperson-oriented model as compared with the models without a history of diabetes or hypertension according to 10-years risk categories meaningful for intervention.

## Figures and Tables

**Table 1:** Baseline characteristics of study cohorts by survey years.

	Cohort				
	1982	1987	1992	1997	2002
No of participants (Men%)	8036 (48.8)	4720 (48.0)	5298 (46.7)	6437 (47.0)	6941 (45.0)
Age, year	43 (11.1)	42 (11.1)	43 (11.3)	44 (11.3)	44 (11.5)
Body mass index, kg/m^2^	25.9 (4.2)	26.1 (4.3)	26.0 (4.4)	26.3 (4.5)	26.6 (4.6)
Systolic blood pressure, mmHg	140 (19.6)	138 (19.2)	135 (19.2)	133 (18.5)	133 (18.7)
Diastolic blood pressure, mmHg,	84 (12.1)	84 (11.6)	81 (12.0)	82 (11.3)	78 (11.5)
HDL-cholesterol, mmol/L	1.31 (1.29)	1.41 (1.29)	1.36 (1.28)	1.37 (1.30)	1.46 (1.31)
Total cholesterol, mmol/L	5.92 (1.23)	5.81 (1.23)	5.50 (1.22)	5.38 (1.21)	5.44 (1.21)
Years of education	9.4 (3.6)	10.3 (3.7)	11.4 (3.8)	12.0 (3.8)	12.8 (3.7)
Current smoker, %	28.8	26.7	28.0	25.4	28.1
History of hypertension, %	12.0	12.4	13.2	15.8	16.1
History of diabetes, %	1.3	1.5	1.6	2.2	2.2
Parent(s) with myocardial infarction, %	29.1	30.3	23.1	21.3	21.4
Incapable of walking 500 meters, %	2.4	1.9	2.1	0.8	1.1
Maximum follow-up years	26.0	21.0	16.0	10.9	6.0
No. of CHD events (%)	945 (11.8)	349 (7.4)	205 (3.9)	141 (2.2)	52 (0.7)

Data are mean (SD) or as noted.

**Table 2:** Baseline characteristics of participants according to incidence of coronary heart disease (CHD) at the end of followup.

	Men		Women	
	Not CHD	CHD	Not CHD	CHD
No. of participants (%)	13684 (92.4)	1131 (7.6)	16056 (96.6)	561 (3.4)
Age, year	43 (11.1)	51 (9.0)	43 (11.3)	55 (8.0)
Body mass index, kg/m^2^	26.5 (3.8)	27.7 (4.0)	25.7 (4.8)	28.7 (5.3)
Systolic blood pressure, mmHg	138 (17.4)	149 (19.1)	132 (19.7)	153 (22.4)
Diastolic blood pressure, mmHg,	84 (11.8)	90 (11.7)	79 (11.3)	87 (11.5)
HDL-cholesterol, mmol/L	1.25 (1.28)	1.15 (1.31)	1.52 (1.22)	1.36 (1.28)
Total cholesterol, mmol/L	5.65 (1.22)	6.39 (1.20)	5.50 (1.22)	6.57 (1.21)
Current smoker, %	35.0	43.9	20.2	20.5
History of hypertension, %	13.4	23.3	12.9	37.1
History of diabetes, %	1.8	5.5	1.3	7.1
Parent(s) with myocardial infarction, %	23.8	28.5	25.4	34.0
Incapable of walking 500 meters, %	1.3	5.1	1.5	8.0
Years of education in tertiles, %				
Low	30.8	36.5	31.0	34.6
Middle	33.4	36.2	32.9	35.8
High	35.8	27.3	36.1	29.6

Data are mean (SD) or percentage.

**Table 3:** Regression coefficients (*β*) and hazard ratio (HR) for incident coronary heart disease estimated based on different Cox models.

	Men (*n* = 14815)		Women (*n* = 16617)	
	*β*	HR (95% CI)	*β*	HR (95% CI)
*Laboratory based *				
Age*, year	3.893	49.07 (35.25–68.32)	4.972	144.38 (78.41–265.82)
Current smoking	0.579	1.78 (1.58–2.01)	0.930	2.53 (2.04–3.15)
History of diabetes	0.950	2.59 (1.99–3.36)	1.185	3.271 (2.33–4.59)
History of hypertension	0.207	1.23 (1.06–1.42)	0.536	1.71 (1.41–2.06)
Incapable of walking 500 meters	0.429	1.54 (1.17–2.01)	0.586	1.80 (1.32–2.45)
Parent(s) with myocardial infarction	0.223	1.25 (1.10–1.42)	0.438	1.55 (1.30–1.85)
Systolic blood pressure*, mmHg	1.491	4.44 (2.74–7.21)	1.933	6.91 (3.66–13.05)
Cholesterol to HDL-C ratio*	1.078	2.94 (2.43–3.55)	0.998	2.71 (2.08–3.54)
Baseline survival function at 10 years				
Cohort 1982	0.978		0.997	
Cohort 1987	0.979		0.996	
Cohort 1992	0.981		0.997	
Cohort 1997–2002	0.984		0.996	

*Non-laboratory based *				
Age*, year	4.012	55.23 (39.76–76.73)	5.256	191.79 (104.26–352.80)
Current smoking	0.630	1.88 (1.66–2.12)	0.956	2.60 (2.10–3.23)
History of diabetes	0.952	2.59 (1.99–3.36)	1.282	3.60 (2.59–5.02)
History of hypertension	0.219	1.24 (1.07–1.44)	0.575	1.78 (1.47–2.14)
Incapable of walking 500 meters	0.480	1.62 (1.23–2.12)	0.539	1.71 (1.25–2.35)
Parent(s) with myocardial infarction	0.258	1.29 (1.13–1.47)	0.402	1.49 (1.25–1.78)
Systolic blood pressure*, mmHg	1.436	4.20 (2.59–6.84)	1.919	6.81 (3.61–12.87)
Body mass index, kg/m^2^	0.951	2.59 (1.65–4.05)	0.732	2.08 (1.26–3.44)
Baseline survival function at 10 years				
Cohort 1982	0.974		0.996	
Cohort 1987	0.977		0.995	
Cohort 1992	0.980		0.997	
Cohort 1997–2002	0.984		0.996	

*Layperson oriented*				
Age*, year	4.219	68.00 (49.20–93.98)	5.739	310.77 (172.55–559.70)
Current smoking	0.642	1.90 (1.68–2.14)	0.929	2.53 (2.04–3.14)
History of diabetes	0.937	2.55 (1.96–3.31)	1.296	3.65 (2.62–5.10)
History of hypertension	0.303	1.35 (1.17–1.57)	0.722	2.06 (1.71–2.47)
Incapable of walking 500 meters	0.462	1.59 (1.21–2.08)	0.491	1.63 (1.19–2.24)
Parent(s) with myocardial infarction,	0.266	1.30 (1.14–1.49)	0.386	1.47 (1.23–1.75)
Body mass index, kg/m^2^	1.147	3.15 (2.02–4.91)	0.887	2.43 (1.47–4.02)
Baseline survival function at 10 years				
Cohort 1982	0.973		0.996	
Cohort 1987	0.977		0.995	
Cohort 1992	0.980		0.997	
Cohort 1997-2002	0.985		0.996	

*Natural logarithm of the continuous variables.

**Table 4:** Reclassifications of individuals based on layperson-oriented model as compared with other two models according to 10-year risk categories meaningful for intervention.

		Layperson oriented				
	<5%	5–9%	10–19%	≥20%	Total	NRI (*P* value)
*Men, n* (%)						
Laboratory based						
Non-events						0.011 (0.001)
<5%	10292 (93.3)	718 (6.5)	13 (0.1)	0 (0.0)	11023	
5–9%	438 (19.6)	1537 (68.6)	264 (11.8)	0 (0.0)	2239	
10–19%	6 (0.7)	333 (37.9)	500 (56.9)	40 (4.6)	879	
≥20%	0 (0.0)	9 (4.6)	97 (50.0)	88 (45.4)	194	
Total	10736	2597	874	128	14335	

Events						0.071 (0.004)
<5%	121 (82.9)	24 (16.4)	1 (0.7)	0 (0.0)	146	
5–9%	22 (13.9)	117 (74.1)	19 (12.0)	0 (0.0)	158	
10–19%	1 (0.8)	43 (33.9)	74 (58.3)	9 (7.1)	127	
≥20%	0 (0.0)	2 (4.1)	19 (38.8)	28 (57.1)	49	
Total	144	186	113	37	480	

Overall NRI						0.081 (0.001)

Non-laboratory based (Layperson oriented + systolic blood pressure)						
Non-events						−0.008 (<0.001)
<5%	10450 (97.2)	301 (2.8)	0 (0.0)	0 (0.0)	10751	
5–9%	286 (11.5)	2085 (84.0)	110 (4.4)	0 (0.0)	2481	
10–19%	0 (0.0)	211 (22.4)	716 (75.9)	16 (1.7)	943	
≥20%	0 (0.0)	0 (0.0)	48 (30.0)	112 (70.0)	160	
Total	10736	2597	874	128	14335	

Events						0.046 (0.018)
<5%	126 (89.4)	15 (10.6)	0 (0.0)	0 (0.0)	141	
5–9%	18 (10.2)	145 (82.4)	13 (7.4)	0 (0.0)	176	
10–19%	0 (0.0)	26 (21.7)	90 (75.0)	4 (3.3)	120	
≥20%	0 (0.0)	0 (0.0)	10 (23.3)	33 (76.7)	43	
Total	144	186	113	37	480	

Overall NRI						0.038 (0.053)

Women, *n* (%)						
Laboratory based						
Non-events						0.005 (<0.001)
<5%	15420 (98.3)	262 (1.7)	6 (0.0)	0 (0.0)	15688	
5–9%	196 (33.3)	331 (56.3)	60 (10.2)	1 (0.2)	588	
10–19%	6 (4.3)	44 (31.4)	76 (54.3)	14 (10.0)	140	
≥20%	0 (0.0)	1 (3.1)	13 (40.6)	18 (56.3)	32	
Total	15622	638	155	33	16448	

Events						0.024 (0.516)
<5%	92 (92.9)	7 (7.1)	0 (0.0)	0 (0.0)	99	
5–9%	12 (29.3)	21 (51.2)	7 (17.1)	1 (2.4)	41	
10–19%	2 (11.1)	4 (22.2)	10 (55.6)	2 (11.1)	18	
≥20%	0 (0.0)	0 (0.0)	3 (27.3)	8 (72.7)	11	
Total	106	32	20	11	169	

Overall NRI						0.029 (0.432)

Non-laboratory based (Layperson-oriented + systolic blood pressure)						
Non-events						−0.006 (<0.001)
<5%	15404 (99.1)	143 (0.9)	0 (0.0)	0 (0.0)	15547	
5–9%	215 (31.1)	449 (64.9)	28 (4.0)	0 (0.0)	692	
10–19%	3 (1.8)	46 (27.7)	113 (68.1)	4 (2.4)	166	
≥20%	0 (0.0)	0 (0.0)	14 (32.6)	29 (67.4)	43	
Total	15622	638	155	33	16448	

Events						0.077 (0.009)
<5%	96 (96.0)	4 (4.0)	0 (0.0)	0 (0.0)	100	
5–9%	9 (29.0)	22 (71.0)	0 (0.0)	0 (0.0)	31	
10–19%	1 (3.8)	6 (23.1)	17 (65.4)	2 (7.7)	26	
≥20%	0 (0.0)	0 (0.0)	3 (25.0)	9 (75.0)	12	
Total	106	32	20	11	169	

Overall NRI						0.071 (0.017)

NRI: net reclassification improvement.
