# Supplementary figures and images for: Layperson-Oriented versus Clinical-Based Models for Assessing 10-Year Incidence of Coronary Heart Disease: National FINRISK Study
Source: Int J Vasc Med. 2011 Oct 19;2011:823782. doi: 10.1155/2011/823782 (PMC3199110; doi:10.1155/2011/823782)

Men

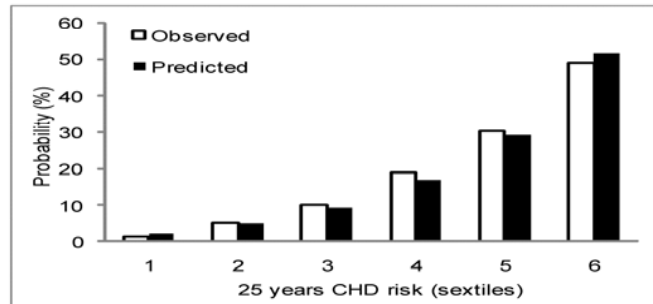

Women

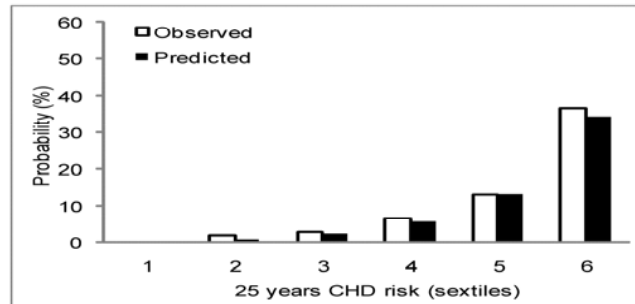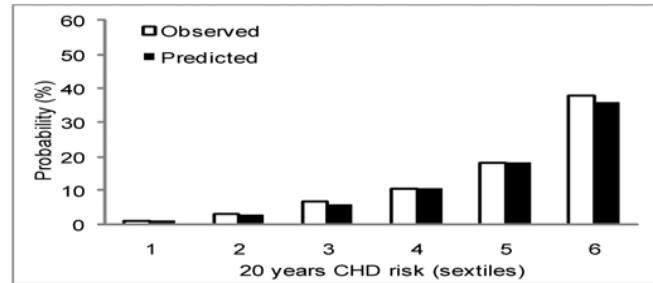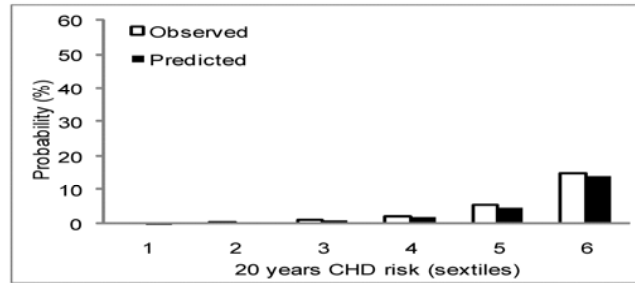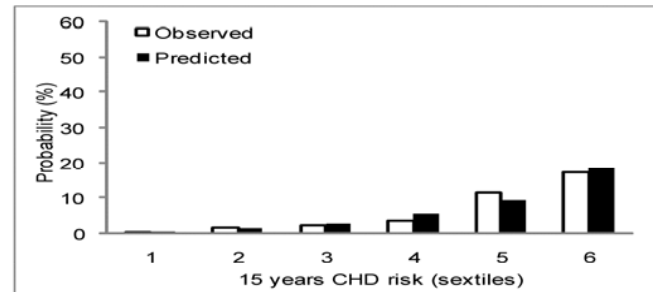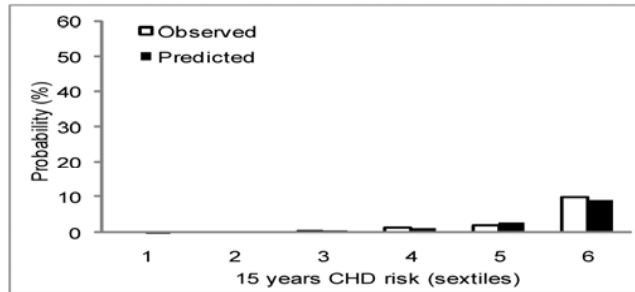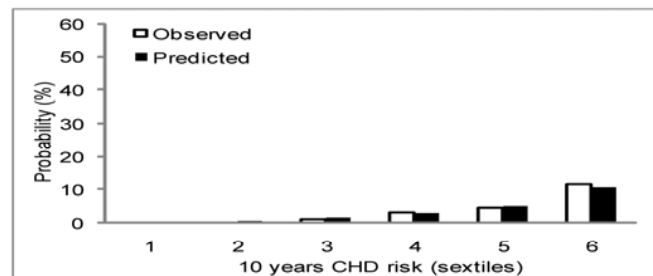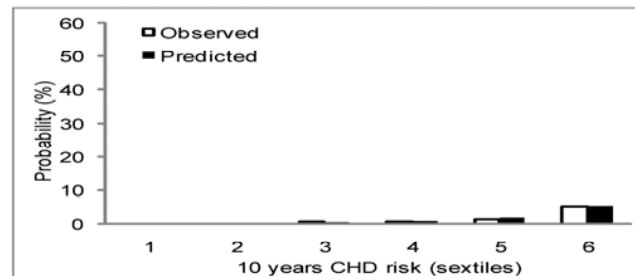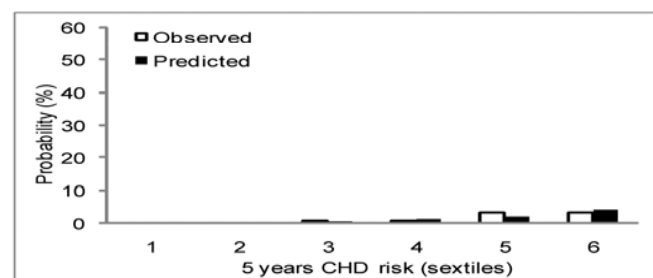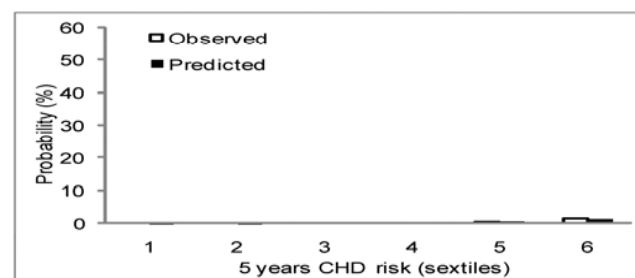

Supplement: Supplementary file 1 — Figure S1. Observed (Kaplan-Meier, white) and predicted (black) probability of coronary heart disease at 5, 10,15, 20 and 25 years of follow-up in each of the six categories of the risk estimated by the layperson-oriented model. Figure S2. Receiver operating characteristic curves for 10-years incidence of coronary heart disease predicted by the layperson-oriented model (blue, AUC=0.803 for men and 0.864 for women), layperson-oriented model without a history of diabetes (green, AUC=0.799 for men and 0.856 for women) or hypertension (red, AUC=0.801 for men and 0.856 for women). The AUC was slightly reduced when the history of diabetes was removed from the layperson model (p=0.04 for both men and women). AUC: Area under the curve. Supplementary Table 1. Variables recorded at the baseline examinations and evaluated in the Cox regression analysis in the current study. Supplementary Table 2. Reclassifications of individuals based on layperson-oriented model as compared with the models without a history of diabetes or hypertension according to 10-years risk categories meaningful for intervention. [file 823782.f1.pdf]

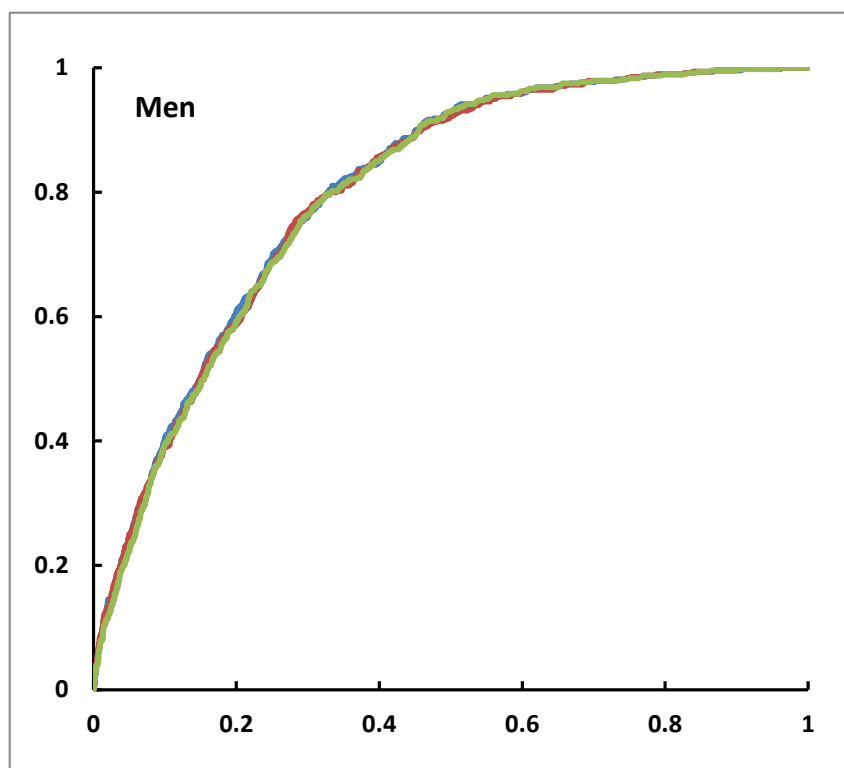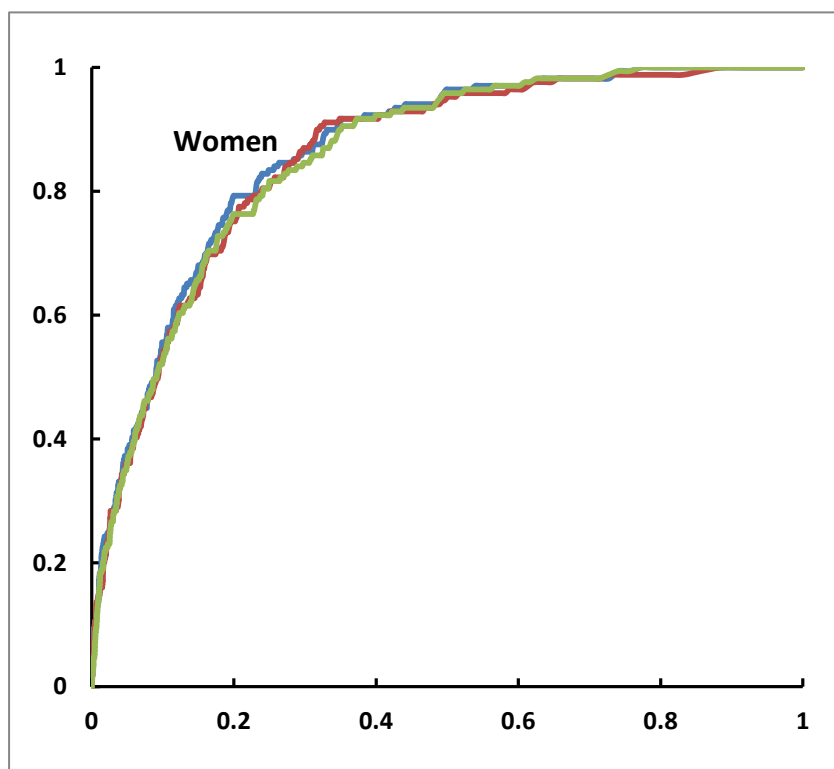

Supplement: Supplementary file 2 [file 823782.f2.pdf]
