# Supplementary material for: Layperson-Oriented versus Clinical-Based Models for Assessing 10-Year Incidence of Coronary Heart Disease: National FINRISK Study
Source: Int J Vasc Med. 2011 Oct 19;2011:823782. doi: 10.1155/2011/823782 (PMC3199110; doi:10.1155/2011/823782)
Supplement: Supplementary file 3 [file 823782.f3.docx]

Supplemental digital table 1. Variables recorded at the baseline examinations and evaluated in the Cox regression analysis in the current study.

| **Variables** | **Description** |
| --- | --- |
| Age, year | Age at the baseline examination |
| Sex | Men; women |
| Blood pressure, mmHg  Systolic blood pressure  Diastolic blood pressure | Mean of the two measurements; measured by a trained nurse from the right arm of a participant after sitting for 5 minutes |
| Body mass index, Kg/m^2^ | weight in kilogram divided by height in square meters |
| Total cholesterol, mmol/l | Enzymatic assay method (CHOD-PAP, Boehringer Mannheim). Before assay HDL-cholesterol was precipitated from the samples by the PTA-precipitation method. Fresh serum samples drawn after 4-hour fasting. |
| HDL cholesterol, mmol/l |  |
| Ratio of cholesterol to HDL (chol/HDL) |  |
| Survey area | Residential cities |
| Cohort | Recruited in 1982, 1987, 1992, 1997, or, 2002. |
| Marital status | Married or cohabited; single; separated or divorced; widow. |
| Education, years | School years |
| Education, years in tertiles | Cohort-specific tertiles of school years |
| History of hypertension | Hypertension confirmed or treated by a doctor last year, yes/no |
| Ever used anti-hypertensive drugs | Yes/no |
| Household’s income last year | Cohort-specific categories of nine |
| History of elevated cholesterol | Ever been told having elevated total cholesterol |
| Present health state (subjective evaluation) | Very good, reasonably good, medium, not very good, very bad |
| Can you do the following without help? | Yes versus No |
| Washing | Washing yourself in most instances |
| Dressing | Get dressed |
| Moving up stairs without stopping | about one-floor higher |
| Walking 500 meters without rest |  |
| Running a short distance | about 100 meters |
| Running a long distance | >500 meters |
| Stress in last month | Almost unbearable; more than usual; no more than usual; no stress at all. |
| Worry about workload | How often do you feel you have to stretch your strength to the extreme in order to cope with your present work or workload?  Almost all the time; quite often; sometimes; seldom; never; I do not work. |
| Stress from work | How often are you troubled by continuous busyness/stress at your work?  Almost all the time; quite often; sometimes; seldom; never; I do not work. |
| Physical activity at work | Sitting work; walking but not lifting heavy things; walking and lifting heavy things; heavy manual labor work. |
| Physical activity in leisure time | No physical activity; low intensive physical activity more than 4 hours per week; moderately intensive physical activity more than 3 hours per week; regular competition training many days a week. |
| History of diabetes | Ever told having diabetes or glucose intolerance by a doctor?  No; glucose intolerance; diabetes. |
| Father with myocardial infarction (MI) or angina pectoris | Had your father been diagnosed as having MI or angina pectoris before age of 60 years?  Yes or no. |
| Mother with MI or angina pectoris | Had your mother been diagnosed as having MI or angina pectoris before age of 60 years?  Yes or no. |
| Father with stroke | Had your father been diagnosed as having stroke before age of 60 years?  Yes or no. |
| Mother with stroke | Had your mother been diagnosed as having stroke before age of 60 years? |
| Smoking status | Never smoking; ex-smokers (smoked regularly but had stopped smoking at least one month before the survey); current smokers (had smoked regularly cigarettes, cigars or pipe for at least one year and had smoked during the preceding month). |
| Type of fat consumed with bread spread | Butter; non- butter |
| Type of cooking oil | Vegetable oil; baking margarine; butter. |
| Type of milk | Full fat milk; low fat milk; fat free milk or do not drink milk. |
| Vegetable consumption, frequency | Not eating vegetable or less than once a month; less than once a day; once or more a day. |
| Fruit consumption, frequency | Not eating fruit or less than once a month; less than once a day; once or more a day. |
| Alcohol consumption, g/week | Any kinds of alcohol consumed; converted to gram per week. |
| Trouble with spouse | Do you have trouble to get along with your spouse?  Almost all the time; quite often; sometimes; seldom; never; I do not have a spouse. |
| Trouble with kids | Have your own children caused you special trouble?  Almost all the time; quite often; sometimes; seldom; never; I do not have a child. |
| What do you think about your marriage or cohabiting? | Very happy; possibly happy; hard to say; quite unhappy; very unhappy; I am not married or do not cohabit. |
| How often are you bothered because your work interferes with your family life? | Almost all the time; quite often; sometimes; seldom; never; I do not have a family or I am not working. |
| Subjective satisfaction with the economic situation | Very satisfied; satisfied; to certain extent satisfied; dissatisfied; very dissatisfied. |
| Subjective satisfaction with the accomplishments in life | Very satisfied; satisfied; to certain extent satisfied; dissatisfied; very dissatisfied. |
| Subjective satisfaction with family life | Very satisfied; satisfied; to certain extent satisfied; dissatisfied; very dissatisfied; I don’t have a family. |
| Is your financial situation better or worse than before? | Much better; a little better; the same as before; a little worse; or much worse. |
| Heart disease can be prevented through lifestyle changes | Completely agree; agree to certain extent; hard to say; disagree to certain extent; completely disagree. |
| Changing diet is not beneficial to the middle-aged people | Completely agree; agree to certain extent; hard to say; disagree to certain extent; completely disagree. |
