# Supplementary material for: Layperson-Oriented versus Clinical-Based Models for Assessing 10-Year Incidence of Coronary Heart Disease: National FINRISK Study
Source: Int J Vasc Med. 2011 Oct 19;2011:823782. doi: 10.1155/2011/823782 (PMC3199110; doi:10.1155/2011/823782)
Supplement: Supplementary file 4 [file 823782.f4.docx]

Supplemental table 2A . Reclassifications of individuals based on layperson-oriented model as compared with the model not requiring history of diabetes according to 10-years CHD risk categories meaningful for intervention .

|  |  | Layperson-oriented model not requiring history of diabetes | | | | | | | | | | |  |  |  |  |  |  |
| --- | --- | --- | --- | --- | --- | --- | --- | --- | --- | --- | --- | --- | --- | --- | --- | --- | --- | --- |
|  |  | <5% | | | 5-9% | | 10-19% | | ≥ 20% | Total | | NRI (p-value) |  |  |  |  |  |  |
| Layperson-oriented model | | | | | | | | | | | | |  |  |  |  |  |  |
| Men, n (%) | | | | | | | | | | | |  |  |  |  |  |  |  |
|  | Non-events |  | |  | |  | |  | | |  | 0.017 (<0.001) |  |  |  |  |  |  |
|  | <5% | 10473 (97.6) | | 263(2.4) | | 0(0.0) | | 0(0.0) | | | 10736 |  |  |  |  |  |  |  |
|  | 5-9% | 45(1.7) | | 2391(92.1 ) | | 161(6.2) | | 0(0.0) | | | 2597 |  |  |  |  |  |  |  |
|  | 10-19% | 12(1.4) | | 92(10.5) | | 743(85.0) | | 27(3.1) | | | 874 |  |  |  |  |  |  |  |
|  | ≥ 20% | 0(0.0) | | 14(10.9) | | 46(35.9) | | 68(53.1) | | | 128 |  |  |  |  |  |  |  |
|  | Total | 10530 | | 2760 | | | 950 | 95 | | | 14335 |  |  |  |  |  |  |  |
|  | Events |  | |  | |  | |  | | |  | 0.025 (0.115) |  |  |  |  |  |  |
|  | <5% | 138(95.8) | | 6(4.2) | | 0(0.0) | | 0(0.0) | | | 144 |  |  |  |  |  |  |  |
|  | 5-9% | 3(1.6) | | 169(90.9) | | 14(7.5) | | 0(0.0) | | | 186 |  |  |  |  |  |  |  |
|  | 10-19% | 1(0.9) | | 13(11.5) | | 96(85.0) | | 3(2.7) | | | 113 |  |  |  |  |  |  |  |
|  | ≥ 20% | 0(0.0) | | 5(13.5) | | 13(35.1) | | 19(51.4) | | | 37 |  |  |  |  |  |  |  |
|  | Total | 142 | | 193 | | 123 | | 22 | | | 480 |  |  |  |  |  |  |  |
| Overall NRI | | | | | | | | | | | | 0.042 (0.009) |  |  |  |  |  | 0.042 (0.009) |
|  | | | | | | | | | | | |  |  |  |  |  |  |  |
| Women, n (%) | | | | | | | | | | | |  |  |  |  |  |  |  |
|  | Non-events |  |  | | |  | |  | | |  | 0.0003 (0.768) |  |  |  |  |  |  |
|  | <5% | 15503(99.2) | 119(0.8) | | | 0(0.0) | | 0(0.0) | | | 15622 |  |  |  |  |  |  |  |
|  | 5-9% | 52(8.2) | 560(87.8) | | | 26(4.1) | | 0(0.0) | | | 638 |  |  |  |  |  |  |  |
|  | 10-19% | 40(25.8) | 25(16.1) | | | 89(57.4) | | 1(0.6) | | | 155 |  |  |  |  |  |  |  |
|  | ≥ 20% | 0(0.0) | 16(48.5) | | | 8(24.2) | | 9(27.3) | | | 33 |  |  |  |  |  |  |  |
|  | Total | 15595 | 720 | | | 123 | | 10 | | | 16448 |  |  |  |  |  |  |  |
|  | Events |  |  | | |  | |  | | |  | 0.047 (0.117) |  |  |  |  |  |  |
|  | <5% | 101(95.3) | 5(4.7) | | | 0(0.0) | | 0(0.0) | | | 106 |  |  |  |  |  |  |  |
|  | 5-9% | 1(3.1) | 28(87.5) | | | 3(9.4) | | 0(0.0) | | | 32 |  |  |  |  |  |  |  |
|  | 10-19% | 4(20.0) | 3(15.0) | | | 12(60.0) | | 1(5.0) | | | 20 |  |  |  |  |  |  |  |
|  | ≥ 20% | 0(0.0) | 6(54.5) | | | 3(27.3) | | 2(18.2) | | | 11 |  |  |  |  |  |  |  |
|  | Total | 106 | 42 | | | 18 | | 3 | | | 169 |  |  |  |  |  |  |  |
| Overall NRI | |  |  | | |  | |  | | |  | 0.048 (0.115) |  |  |  |  |  |  |

Supplemental table 2B . Reclassifications of individuals based on layperson-oriented model as compared with the model not requiring history of hypertension according to 10-years CHD risk categories meaningful for intervention .

|  | | | Layperson-oriented model not requiring history of hypertension | | | | | | | | | | |
| --- | --- | --- | --- | --- | --- | --- | --- | --- | --- | --- | --- | --- | --- |
|  | |  | | <5% | | 5-9% | | 10-19% | | ≥ 20% | Total | NRI (p-value) |  |
| Layperson-oriented model  Men, n (%) | | | | | | | | | | | |  | |
|  | Non-events | |  | |  | |  | |  | |  | 0.008 (<0.001) | |
|  | <5% | | 10441(97.3) | | 295(2.7) | | 0(0.0) | | 0(0.0) | | 10736 |  | |
|  | 5-9% | | 162(6.2) | | 2307(88.8) | | 128(4.9) | | 0(0.0) | | 2597 |  | |
|  | 10-19% | | 0(0.0) | | 138(15.8) | | 715(81.8) | | 21(2.4) | | 874 |  | |
|  | ≥ 20% | | 0(0.0) | | 0(0.0) | | 29(22.7) | | 99(77.3) | | 128 |  | |
|  | Total | | 10603 | | 2740 | | 872 | | 120 | | 14335 |  | |
|  | Events | |  | |  | |  | |  | |  | -0.002 (0.906) | |
|  | <5% | | 132(91.7) | | 12(8.3) | | 0(0.0) | | 0(0.0) | | 144 |  | |
|  | 5-9% | | 12(6.5) | | 158(84.9) | | 16(8.6) | | 0(0.0) | | 186 |  | |
|  | 10-19% | | 0(0.0) | | 17(15.0) | | 88(77.9) | | 8(7.1) | | 113 |  | |
|  | ≥ 20% | | 0(0.0) | | 0(0.0) | | 6(16.2) | | 31(83.8) | | 37 |  | |
|  | Total | | 144 | | 187 | | 110 | | 39 | | 480 |  | |
| Overall NRI | | | | | | | | | | | | 0.006 (0.737) | |
|  | | | | | | | | | | | |  | |
| Women, n(%) | | | | | | | | | | | |  | |
|  | Non-events | |  | |  | |  | |  | |  | -0.013 (<0.001) | |
|  | <5% | | 15459(99.0) | | 163(1.0) | | 0(0.0) | | 0(0.0) | | 15622 |  | |
|  | 5-9% | | 338(53.0) | | 257(40.3) | | 43(6.7) | | 0(0.0) | | 638 |  | |
|  | 10-19% | | 0(0.0) | | 80(51.6) | | 67(43.2) | | 8(5.2) | | 155 |  | |
|  | ≥ 20% | | 0(0.0) | | 0(0.0) | | 15(45.5) | | 18(54.5) | | 33 |  | |
|  | Total | | 15797 | | 500 | | 125 | | 26 | | 16448 |  | |
|  | Events | |  | |  | |  | |  | |  | 0.118 (0.001) | |
|  | <5% | | 99(93.4) | | 7(6.6) | | 0(0) | | 0(0) | | 106 |  | |
|  | 5-9% | | 16(50.0) | | 14(43.8) | | 2(6.3) | | 0(0.0) | | 32 |  | |
|  | 10-19% | | 0(0.0) | | 9(45.0) | | 11(55.0) | | 0(0.0) | | 20 |  | |
|  | ≥ 20% | | 0(0.0) | | 0(0.0) | | 4(36.4) | | 7(63.6) | | 11 |  | |
|  | Total | | 115 | | 30 | | 17 | | 7 | | 169 |  | |
| Overall NRI | | |  | |  | |  | |  | |  | 0.105 (0.004) | |

NRI, net reclassification improvement
